# A Correlative Study of Sunflower Seed Vigor Components as Related to Genetic Background

**DOI:** 10.3390/plants9030386

**Published:** 2020-03-20

**Authors:** Marine Saux, Benoît Bleys, Thierry André, Christophe Bailly, Hayat El-Maarouf-Bouteau

**Affiliations:** 1 Sorbonne Université, CNRS, Laboratoire de Biologie du Développement, F-75005 Paris, France, christophe.bailly@sorbonne-universite.fr (C.B.); 2 Soltis, 6 chemin de Panedautes, 31700 Mondonville, France, thierry.andre@soltis-research.com (T.A.)

**Keywords:** seed, vigour, genotype

## Abstract

Seed vigor is an important trait that determines seed performance in the field, which corresponds to seed germination rate and seedling establishment. Previous works brought helpful equations to calculate several parameters allowing vigor characterization. In this work we used base water potential (Ψb), base temperature (Tb) and seed lot (Ki) constants to characterize the vigor of 44 sunflower seed lots. Contrasting responses to water or temperature stress and storage potential were recorded within this population, the most interesting being the opposite responses between Ψb and Ki. The genotypes that were resistant to water stress presented low ability for storage and *vice versa*. Furthermore, Ψb and Ki presented narrow ranges while Tb showed important variability within the 44 genotypes. The analysis of the whole dataset showed that these constants are not correlated to each other or to the seed size, suggesting that genetic background is the most important determining factor in seed performance. Consequently, vigor characterization of genotypes is needed in the crop selection process in order to optimize agricultural productivity.

## 1. Introduction:

Seed germination, vigor and viability describe different aspects of the quality of a seed population, and these three aspects need to be addressed together to understand the overall quality of a seed lot. Therefore, results of seed germination tests must be analyzed together with results of seed vigor tests in order to differentiate seed lots of acceptable germination [1]. Germination is defined as the protrusion of the radicle through the seed envelopes in favorable conditions. However, germination environments can be far from favorable and can impose stresses on seeds, which can delay or prevent germination. The standard germination assay performed in the laboratory is thus a poor predictor of emergence in non-optimal environments. Seed vigor reflects properties of a seed to germinate in a wide range of environmental conditions. It can be defined as the capacity of seeds to lead to rapid and homogenous seedling emergence and stand establishment [2]. Since crop yield is a function of plant density, seed vigor can influence crop yield by its effects on emergence. High seed vigor may increase crop yield [1]. If seed quality only altered emergence percentage, then growers could overcome such effects by adjusting sowing rates. However, in practice, seed quality is affected by the seedbed environment, which make it is difficult to adjust and predict seed sowing rates [3]. At last, seed viability refers to whether or not a seed contains any metabolically active tissues and enzymes capable of sustaining living plant cells.

When seed environmental conditions are close to optimum at the time of sowing, field emergence can correlate with germination test results. However, such conditions are not often encountered in practice, and field conditions are often sub-optimal, which may lead to different field performance depending on the vigor status of the seed lot. This may cause difference in emergence level, rate and growth uniformity and sometimes lower vegetative and reproductive yield. Thus, high vigor seed lots are expected to perform better under environmentally stressed conditions than low vigor seed lots, even if standard germination test results can be comparable (International Seed Testing Association (ISTA)) [4]. Since then, the ISTA [4,5] brought a more specific definition that takes into account not only those properties that determine the activity and level of performance of seed lots of acceptable germination in a wide range of environments but also the performance after storage.

Many environmental factors influence germination, temperature (T) and water potential (ψ) being the most critical [6,7]. The thermal time (θ_T_) approach has been used to characterize the time to germination at different temperatures and, by analogy, the hydrotime describes the relationship between ψ and seed germination [8]. Mathematical models describing germination patterns in response to T and ψ have been developed (discussed in [9]). 

The genetic basis of seed vigor has been established, although it is poorly understood [10]. In the last decades, molecular aspects of seed dormancy and germination have been reported in different species (for review [11,12,13]) and in sunflower in particular [14,15,16,17]. Several genes have been related to seed vigor [18,19,20], but, they can hardly be used as vigor genetic markers. The quantitative trait loci (QTL) approach allows the identification of loci that influence seed vigor in *Brassica oleracea*, such as speed of germination (SOG1), which contains two genes related to abscisic acid that influence negatively the speed of germination [21,22,23]. Speed of germination is nevertheless one property among others in the characterization of seed vigor, which is a complex trait, and its proper evaluation is highly challenging. In fact, the causes of difference in seed vigor can come from the genotype, the nutrition and growth conditions of the mother plant, the physiological maturity of the seed at harvest, the physical handling of the seed during processing, seed moisture content and the temperature during storage [10].

In this paper, we investigated sunflower (*Helianthus annuus*) seed vigor in 44 genotypes produced simultaneously in the same field in order to overcome the differences due to the environment. Seed germination was assessed in response to water and temperature stress and after accelerated ageing, and the corresponding constants and their relevance in seed vigor were determined. Our objectives were to decipher the relationship between the various components of seed vigor and to determine whether they are controlled by the genetic background.

## 2. Results

### 2.1. Effect of Water Stress

**Figure 1:**
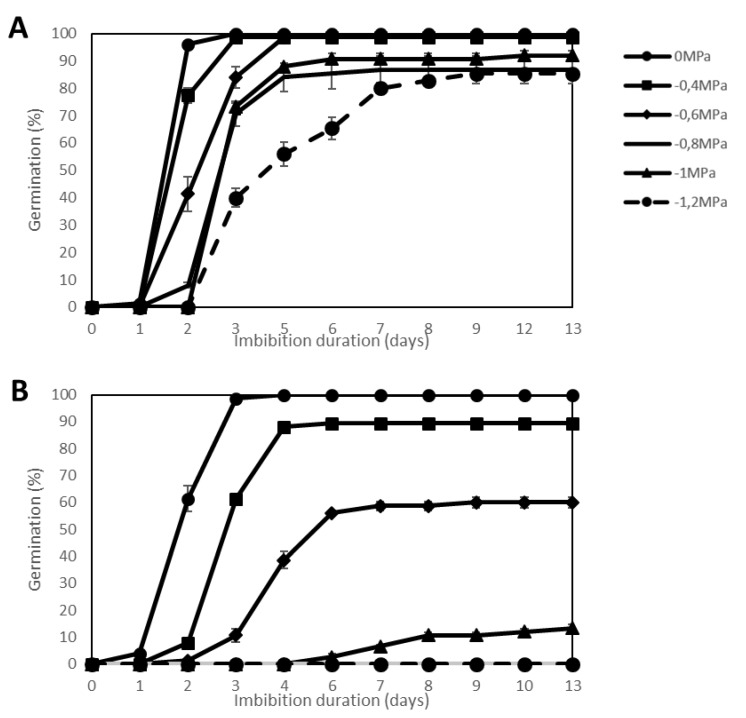
Germination curves of 2 sunflower genotypes, *Helianthus annuus* sp. SOLQUA-160 (**A**) and SOLQUA-165 (**B**) Water stress (−0.4 MPa; −0.6 MPa; −0.8 MPa; −1 MPa; −1.2 MPa) was applied during germination at 20 °C in comparison to the control on water (0 MPa).

Several intensities of water stress (from −0.2 to −1.2 MPa) were applied to the seeds of 44 genotypes using PEG solutions instead of water (0 MPa) during germination at 20 °C, and kinetics of germination were recorded (Table S1). As an example, Figure 1 shows contrasting germination behavior of the 2 genotypes *SOLQUA*-160 (Figure 1A) and *SOLQUA*-165 (Figure 1B). At 20 °C, seeds of both genotypes fully germinated within 3 d at 20 °C (Figure 1). Decreasing water availability by increasing PEG concentrations in the imbibition media had an inhibitory effect on seed germination. Seeds of *SOLQUA*-165 were more sensitive to water stress as their germination could not reach 100% when water stress was applied even at low PEG concentrations. At high stress (−1.2 MPa), seeds of SOLQUA 165 were unable to germinate (Figure 1B), whereas seeds of *SOLQUA*-160 reached at least 80% of germination (Figure 1A).

### 2.2. Effect of Temperature Stress

**Figure 2:**
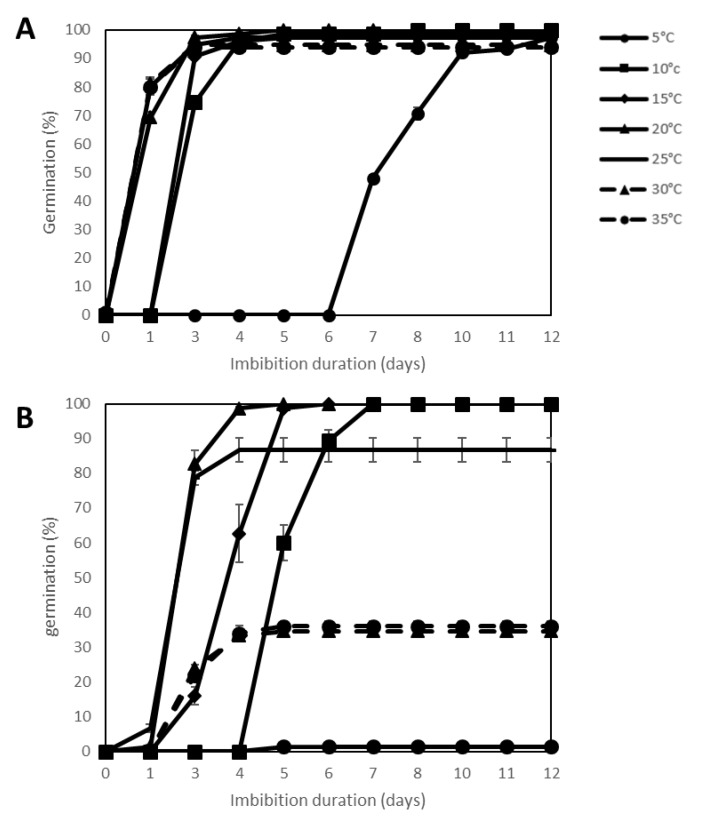
Germination curves of 2 sunflower genotypes, *Helianthus annuus* sp. SOLQUA-160 (**A**) and SOLQUA-165 (**B**) Temperature stress (5, 10, 15, 25, 30 and 35 °C) was applied during germination in comparison to the control on water at 20 °C.

Several temperatures (5, 10, 15, 20, 25, 30 and 35 °C) were applied to the seeds of the 44 genotypes during germination tests on water (Table S1). Figure 2, comparing germination percentages of seeds of *SOLQUA-160* and *SOLQUA-165*, shows that both genotypes can germinate to 100% between 10 and 25 °C, which correspond to optimal temperatures for sunflower seed germination. However, for high temperatures such as 30 and 35 °C *SOLQUA-165* seed germination was reduced to less than 40% and for low temperature (5 °C), their germination was arrested (Figure 2B). In contrast, even if the rate of germination of *SOLQUA-160* seeds was lowered at 5 °C, their final germination percentage reached 100% at all temperatures tested (Figure 2A).

### 2.3. Effect of Accelerated Ageing

**Figure 3:**
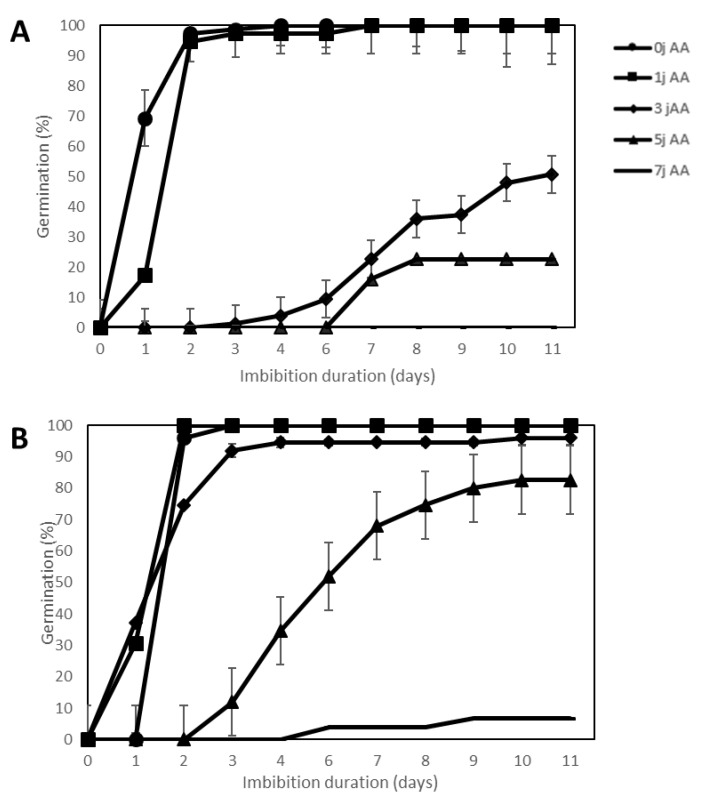
Germination curves of 2 sunflower genotypes, *Helianthus annuus* sp. SOLQUA-160 (**A**) and SOLQUA-165 (**B**) Accelerated ageing (AA) was applied for 1, 3, 4 or 7 days (as compared to the control 0, which corresponded to non-aged seeds); germination tests were then performed on water at 20 °C.

Accelerated ageing (100% RH and 45 °C) was applied for 1, 3, 4 or 7 days as compared to the control 0, which corresponded to non-aged seeds. This treatment first decreased seed germination speed then seed viability as estimated by the final germination percentage after 11 d at 20 °C (Figure 3). Figure 3 shows that seeds of *SOLQUA-160*, which were quite tolerant to water and temperature stresses, were more sensitive to ageing when compared to seeds of *SOLQUA-165*. In fact, 3 or 5 days of treatments decreased germination percentages of seeds of *SOLQUA-160* to around 50 and 20%, respectively, while *SOLQUA-165* seeds germinated to 95 and 82% in the same conditions (Figure 3).

### 2.4. Determination of Seed Vigor Constants

Using the mathematic models described by Ellis and Roberts [24] and Bradford [8], we calculated the seed vigor parameters, i.e., base water potential (Ψb), base temperature (Tb) and the viability constant (Ki) of the 44 genotypes (Table 1). Three cardinal temperatures corresponding to the base (*T*_b_), optimum (*T*_o_) and maximum temperatures (*T*_c_) were determined for germination [25]. They are useful to determine the best planting period for a given specie. We focused on Tb because sunflower seed vigor was expressed at low temperatures, and the purpose of the present study was to characterize germination parameters in suboptimal conditions of germination. Tb, which represented the lower thermal limit for seed germination, ranging from negative values as low as −3 °C to 5.62 °C according to the genotype (Table 1). Variability of seed response to water stress was shown by Ψb values, which also differed markedly among genotypes (Table 1). Ki value represented the initial quality of the seedlot and was determined using germination values after accelerated ageing; the higher it is, the higher is the initial quality [24]. As for the other vigor constants studied here, our analysis highlighted a large variability of Ki values among seed lots; they ranged from 4.2 to 1.1 (Table 1). In order to analyze the correlation that may exist between Ψb, Tb and Ki, corresponding values were analyzed using the Pearson correlation method. Table 2 shows very low R^2^ (between 0.035 and 0.058), indicating that these parameters were not correlated to each other (Table 2). 

**Figure 4:**
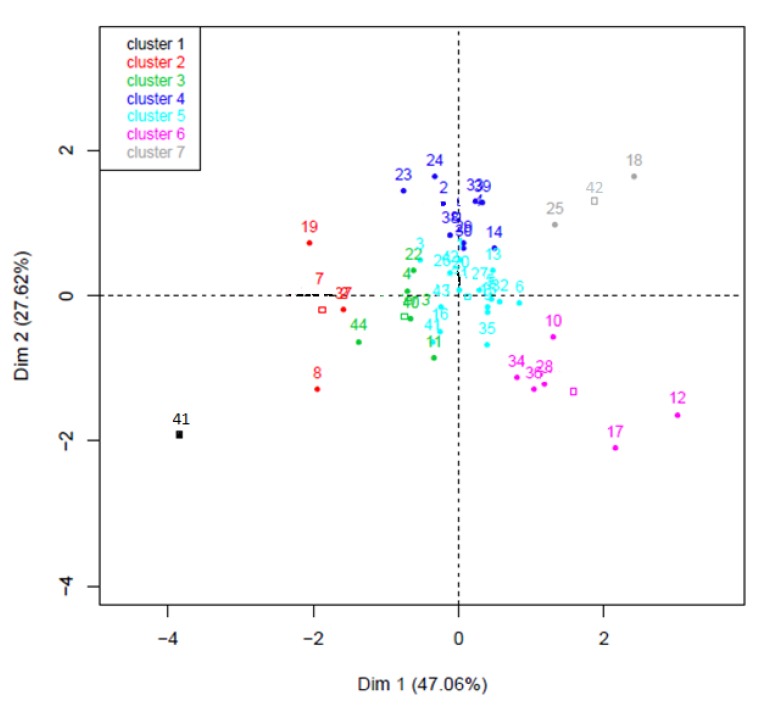
Hierarchical clustering on principal components analysis using seed trait parameters Ψb, Tb and Ki of the 44 genotypes. Genotypes are represented by numbers, according to Table 1.

We next performed a principal component analysis (PCA) using the seed vigor constants, which permitted clusterization of the genotypes (Figure 4, Table 3). From this analysis, thresholds values of Ψb of −1.1 MPa and of Ki of 1.5 were determined. Tolerant seed lots to water stress displayed a Ψb < −1.1 and seeds of high initial quality that may be tolerant for storage of Ki > 1.5. The analysis allowed the distribution of genotypes with a high PCA percentage (74,68%) in seven clusters corresponding to marked resistance or sensitivity to one or several stresses (Figure 4, Table 3). The cluster 5 contained an important number of genotypes (17), which did not show a marked response to extreme conditions (Table 3). In the other clusters, genotypes displayed contrasted responses. For example, cluster 6 regrouped genotypes that presented high initial quality, tolerance to low temperature and sensitivity to water stress, while cluster 7 included those that presented the same responses but were also sensitive to high temperatures. Interestingly, these clusters of high initial quality were sensitive to water stress when clusters from 1 to 4 grouped seed lots of low initial quality that were tolerant to water stress (Table 3). On the other hand, a very small number of genotypes were represented in cluster 1 and 7 (Table 2). However, in these clusters one can find the interesting genotypes presented earlier, namely *SOLQUA-160* in the cluster 1 and *SOLQUA-165* in the cluster 7 (Figure 1, Figure 2 and Figure 3); i.e., in two opposite sides of the PCA (Figure 4). Furthermore, the seed lots of high and low 1000 SW were represented in all the clusters without distinctive distribution (Table 3). 

**Figure 5:**
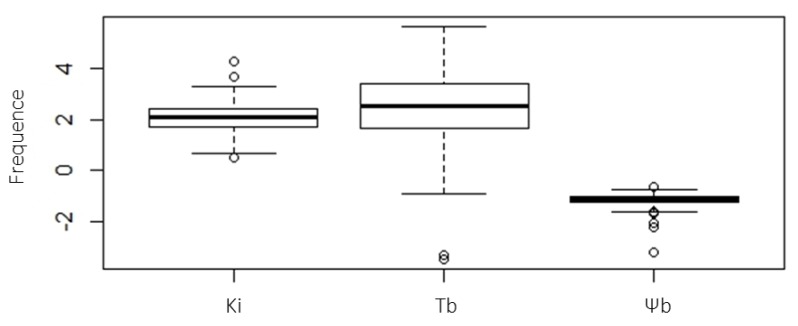
Boxplot presentation of frequency statistical analysis of Ψb, Tb and Ki values of the 44 genotypes.

Lastly, in order to analyze the impact of each stress (temperature, water and ageing) on the 44 genotypes, we displayed the data sets of Ki, Tb and Ψb as a boxplot to show the frequency distribution of the data (Figure 5). Figure 5 shows the small variability of Ki and Ψb values as shown by the width of the corresponding boxes, even though there are some outliers. Tb, however, showed important variability within the 44 genotypes (Figure 5). 

## 3. Discussion

In optimal conditions (germination in water at 20 °C), seeds of the 44 sunflower genotypes studied here were able to germinate to 100% (Table S1). These conditions nevertheless rarely correspond to those encountered in the field where temperature and water potential can vary continuously. When subjected to water stress, temperature stress or ageing treatment, seeds show different capacities to face one or several stresses. This fact was well illustrated by germination tests of *SOLQUA 160* and *SOLQUA 165* seeds, which presented contrasting responses to stress conditions (Figure 1, Figure 2 and Figure 3). 

To study the importance of each component of seed performance (resistance to water stress, temperature stress and ageing), Ψb, Tb and Ki were calculated and analyzed. This analysis allowed a fine characterization of the 44 genotypes, which can be useful for the breeder, as seed vigor has an economic impact for sale but also for seed multiplication [10]. It is worth noting that these parameters can be used to determine a threshold for genotype classification as sensitive or resistant to water stress or low or high initial quality, which is more complicated for temperature response as seed performance can concern low or high temperatures. Using these parameters, further characterization of these 44 seed lots has been performed using PCA (Figure 4). The clustering allowed classification based on seed lot response to temperature or water stresses and initial quality (Table 3). Seventeen out of 44 (cluster 5) seed lots did not show marked tolerance or sensitivity to one or more vigor parameters suggesting that more than 38% of these selected lines, which are representative of genetic variability within Soltis, did not display important vigor. Clusters 2, 3 and 4 contain genotypes (18/44) presenting low initial quality but good tolerance to water stress at the same time (Table 1 and Table 3). The cluster 6 is represented by six genotypes that are classified as tolerant to low temperature and sensitive to water stress with high initial quality (Table 3). Therefore, it is worth noting that tolerance to water stress is associated with low initial quality and vice versa in all genotypes subjected to non-optimal conditions. Regarding the response to temperature, the 44 seed lots present a wide range of Tb as shown by the box plot analysis (Figure 5). Castillo-Lorenzo et al. [26] have reported that Tb range can be narrow in five *H. annuus* genotypes that differ in their flowering time and seed oil composition without correlation with seed oil content. In our study, no correlation was found either between Tb and the other vigor parameters (Table 2), or with seed oil content (data not shown), unlike Gonzales Belo et al. [27], who reported contradictory findings. Ki, calculated using accelerated ageing treatments, can also be used to compare seed lots for their germination performance after storage [25]. Seed initial quality determination being closely related to seed response to high temperature and humidity, it can be assumed that the Ki takes into account the sensitivity to temperature out of context of accelerated ageing; however, one can notice that initial quality or water stress are not necessarily related to response to high temperatures. However, tolerance to low or high temperature represents important information for optimization of sunflower cultivation (Table 3). 

Among the 19 genotypes that are tolerant to water stress with high initial quality (clusters 1 to 4), 9 have a 1000 SW > 50 (high) and 10 have a 1000 SW < 50 (low) (Table 3). In the opposite category (clusters 5 and 6), the proportion of high and low 1000 SW is 5 vs. 3, respectively (Table 3). In the literature, the correlation between seed size or weight and vigor is controversial even though seed size is generally positively correlated with vigorous seedlings and field performance [28]. Previous works on wheat have shown that grain weight or size had no significant effect on germination [29,30], while other papers have reported that seed size was correlated with final seed germination and vigorous seedlings [31,32]. In sunflower it has been shown that the effect of seed size was significant on germination percentage, the germination of large seeds being less important [33,34]. Our results show that there is no correlation between vigor parameters and seed weight in sunflower. Among the 44 genotypes, maintainers (23/44) had high 1000 SW (>50) and restorers (21/44) had mostly low 1000 SW (only 3 had a 1000 SW > 50) (B and R lines, respectively, Table S1). Such difference can be explained by the fact that maintainer and restorer lines belong to 2 genotype groups corresponding to plants characterized by one or several capitula, respectively (Soltis personal data), suggesting that the size is correlated to the genotype but not to vigor parameters. 

Based on genetic distance (Soltis personal data), within each cluster, the closer the genotypes, the closer are their values of vigor parameters. For example, in cluster 2, *SOLQUA-168* (R) and *SOLQUA-068-5* (R), which are more closely related with each other than with *SOLQUA-031* (B), had almost the same Ψb (−1.6) and Ki (1.4) values, while *SOLQUA-031* had a Ψb of −2.2 and a Ki of 2.1 (Tb was variable but high for all of them). On the other hand, even if some closely related genotypes belonged to different clusters, they could have close Ψb and Ki values, such as *SOLQUA-68-5* (cluster 2) and *SOLQUA-110* (cluster 3) that displayed Ψb and Ki values of −1.6, 1.4 and −1.1, 1.1, respectively (Table 1 and Table S1). Less frequently, some closely related genotypes may have different Ψb, Tb and Ki, such as *SOLQUA-132* and *SOLQUA-133* (Table 1 and Table S1), indicating that genetic variation, as small as it may be, can induce physiological changes, especially when characterizing vigor, which encompasses many physiological processes. In fact, to meet all the criteria, the seed must possess the mechanisms of tolerance to water and thermal stress in addition to having mechanisms of protection against ageing. This demonstrates that genotype characterization with vigor parameters is definitely necessary to make the best selection.

Controversial findings about the correlation between seed traits, such as seed size or oil composition, and vigor parameters highlight the important part of the genetics that can be, in addition, significantly influenced by the environment (temperature, water and oxygen) from fertilization to seedling emergence [10]. Being unable to control the environmental conditions, it is important to have relevant tests to predict seed performance. Here we show that sunflower seeds display contrasting responses to water stress and seed initial quality. Consequently, Ψb and Ki may represent the parameters of importance in relation to seed response to unfavorable environmental conditions. Tb can also be of high interest for breeders or farmers to choose the suitable cultivation areas according to local temperatures. This indicates how hard it is to get vigorous seed lots that can meet all the criteria assigned to vigor, but these are important in the characterization of genotypes in the selection process.

## 4. Materials and Methods

### 4.1. Plant Material

Experiments were carried out with seeds of 44 sunflower (*Helianthus annuus* L.) lines corresponding to different genotypes used in sunflower breeding program by SOLTIS (Table S1). They correspond to 21 restorer lines called “R” characterized by small seed size (mean weight of 1000 seeds per g (1000 SW) < 50) and 23 maintainer lines called “B” characterized by large seeds (1000 SW > 50). The 1000 SW were provided by Soltis (Table S1). They were produced in 2014 in the same field in Occitanie, France. Seeds were stored at 20 °C and 60% relative humidity (RH) for three months in order to break dormancy and then kept at 10 °C during the experiment time. 

### 4.2. Seed Treatments and Germination

Germination assays were performed in darkness at 20 °C in 10 cm petri dishes by placing whole achenes (25 per dish, 3 replicates) on a layer of cotton wool moistened with distilled water or with a range of polyethylene glycol (PEG) solutions giving water potential values from −0.4 to −1.2 MPa [35]. The effect of temperature on germination was also analyzed by placing seeds on water in a range of temperature from 5 to 35 °C. Seed accelerated ageing was performed using 100% RH and 45 °C as storage conditions for 24, 72, 120 and 168 h. After ageing, seed germination was evaluated on water at 20 °C for 10 days. A seed was considered germinated when the radicle had pierced the envelopes (seed coat + pericarp). Germination counts were made daily for 11 to 13 days. 

### 4.3. Germination Parameter Calculation

4.3.1. t50 Calculation

The time to obtain 50% germination (t50) was calculated according to the following formula of [36], as modified by [37]:

t50 = ti + ((N/2 − ni)(ti − tj)]/ni − nj(1)

where N is the final number of germinating seeds, and *nj* and *ni* are the cumulative number of seeds germinated by adjacent counts at times *tj* and *ti*, respectively, when *ni < N/2 < nj.*

#### 4.3.2. Base Water Potential

Germination results were analyzed using the hydrotime model described by Bradford [38], which allowed the calculation of the base water potential Ψb, which corresponds to the minimum Ψ that allows germination [8]. The following equations describe the basis of the hydrotime model:

ΘH = (Ψ − Ψb(g)) × tg(2)

GRg = 1/tg = (T − Tb)/ΘT(g) = (Ψ − Ψb(g))/ΘH(3)

Probit(g) = (Ψ − (ΘH/tg) − Ψb (50))/σ Ψb(4)

where ΘH is the hydrotime constant (Hydrotime); ΘT(g) is the time constant function of temperature permitting radicle emergence of percentage g of seed population; Ψ is the water potential of the water source (MPa); Ψb is the base, or minimum, water potential permitting germination (MPa); tg is time to radicle emergence of germination percentage *g* (h); T is imbibition temperature; Tb is the minimum temperature allowing radicle emergence; GRg is the germination rate of percentage *g* of the seed population (h^−1^); σ Ψb is the standard deviation in base water potential within the seed population (MPa).

#### 4.3.3. Base Temperature

The minimum or base temperature (Tb) is the lowest T at which germination can occur [8]. It is calculated using the following formula:

θT(g) = (T − Tb)tg, or GRg = 1/tg = (T − Tb)/θT(g)(5)

ΘT(g) = (T − Tb) × tg(6)

GRg = 1/tg = (T − Tb)/ΘT(g)(7)

Probit(g) = log((T − Tb) × tg) − log ΘT(50)/σ ΘT(8)

where ΘT(g) is the time constant function of temperature permitting radicle emergence of g percentage of the seed population; tg is the time to radicle emergence of germination percentage *g* (h); T is the imbibition temperature; Tb is the minimum temperature allowing radicle emergence; GRg is the germination rate of percentage *g* of the seed population (h^−1^); σ ΘT is the standard deviation of the base temperature. 

This model predicts that the germination rate for a given seed fraction or percentage g (GRg or 1/tg) is a linear function of T above Tb, with a slope of 1/θT (g) and an intercept on the T axis of Tb.

#### 4.3.4. Viability Constant Calculation (Ki)

Viability constant (Ki) permits one to evaluate the initial quality of a seed lot before the ageing process. The equation of this constant as follows [24]:

ν = Ki − p/σ(9)

where ν is viability after *p* days in storage (probit); p is the storage period (days); Ki is the seed-lot constant probit percentage viability at the beginning of storage; σ is the slope of the line, which shows germination percentage (in probit) function of storage duration. 

Ki is obtained after a probit analysis of a mortality curve of a seed lot obtained after accelerating ageing. This regression analysis follows the mortality frequencies of a seed lot across time (days). Ki is the intercept of the regression with the slope v with y axes of a seed lot.

### 4.4. Statistical Analyses

Matrix correlation calculation, PCA, ANOVA and box plot analyses were performed using FactoMineR package in R software.

## Figures and Tables

**Table 1:** Seed vigor parameters, base water potential (Ψb, 50%), base temperature (Tb) and viability constant (Ki) of seeds of the 44 genotypes. nd: non determined.

Number	Genotypes	Ψb (50%) (MPa)	Tb (°C)	Ki
1	*SOLQUA-003*	−1.066	3.07	2.0823
2	*SOLQUA-006*	−1.2284	2.15	2.3716
3	*SOLQUA-007*	−1.1135	1.58	2.3209
4	*SOLQUA-011*	−0.9311	4.53	2.0814
5	*SOLQUA-015-3*	−0.9404	4.17	2.4945
6	*SOLQUA-020*	−1.0896	3.62	1.6434
7	*SOLQUA-023*	−0.8251	3.06	0.7898
8	*SOLQUA-024*	−0.9446	1.72	1.9408
9	*SOLQUA-028*	−0.9856	1.27	2.3179
10	*SOLQUA-030*	−1.0049	−0.9	2.034
11	*SOLQUA-031*	−2.217	4.39	2.169
12	*SOLQUA-037*	−1.0511	2.4	1.8161
13	*SOLQUA-037-2*	−1.1699	−0.36	2.0397
14	*SOLQUA-040*	−2.0158	2.27	1.0646
15	*SOLQUA-047*	−1.1548	5.42	2.0977
16	*SOLQUA-048*	−0.9483	1.5	1.8038
17	*SOLQUA-050*	−0.9966	2.93	1.6187
18	*SOLQUA-055*	−1.1191	0.05	2.6992
19	*SOLQUA-056-3*	−1.1189	0.79	1.8438
20	*SOLQUA-056-4*	−1.1355	−0.86	2.0945
21	*SOLQUA-057*	−1.1903	1.23	1.2352
22	*SOLQUA-068-5*	−1.6805	3.67	1.4013
23	*SOLQUA-073*	−0.966	3.76	1.9841
24	*SOLQUA-075*	−0.9354	−3.44	3.2565
25	*SOLQUA-088*	−1.1602	4.08	2.9622
26	*SOLQUA-096*	−1.2488	5.33	2.7511
27	*SOLQUA-107*	−1.0686	2.38	2.3946
28	*SOLQUA-109*	−1.1588	2.57	3.6534
29	*SOLQUA-110*	−1.1102	2.42	1.1141
30	*SOLQUA-110-2*	−1.5864	1.69	nd
31	*SOLQUA-113*	−0.9967	2.86	2.4624
32	*SOLQUA-114*	−0.9953	3.39	2.1343
33	*SOLQUA-123*	−1.2608	3.33	2.5701
34	*SOLQUA-127*	−1.2095	2.64	0.4968
35	*SOLQUA-132*	−0.6323	3.4	0.6833
36	*SOLQUA-133*	−1.3559	2.95	2.373
37	*SOLQUA-138*	−1.2303	1.67	2.3454
38	*SOLQUA-143*	−1.3089	1.78	1.7256
39	*SOLQUA-147*	−1.244	2.29	1.8077
40	*SOLQUA-148*	−1.1306	−3.3	2.6047
41	*SOLQUA-160*	−3.1991	3.35	1.1666
42	*SOLQUA-165*	−0.7507	2.5	4.2442
43	*SOLQUA-168*	−1.6257	5.62	1.4051
44	*SOLQUA-169*	−1.094	2.64	1.9944

**Table 2:** Correlation between Ψb, Tb and Ki (R^2^).

Variables	Ψb (50%) (MPa)	Tb (°C)	Ki
Ψb (50%) (MPa)	1 (p = 0)	0.035 (p = 0.226)	0.058 (p = 0.116)
Tb (°C)	0.035 (p = 0.226)	1 (p = 0)	0.036 (p = 0.218)
Ki	0.058 (p = 0.116)	0.036 (p = 0.218)	1 (p = 0)

**Table 3:** Description of genotypes belonging to the different clusters; 1000 SW corresponds to the weight of 1000 seeds (g).

Cluster Number	Description of Seed Lot Response	Number of Genotypes	Number of Genotypes Whose 1000 SW > 50	Number of Genotypes Whose 1000 SW < 50
1	Tolerant to high temperatures Tolerant to water stress Low initial quality	1	0	1
2	Tolerant to low temperatures Tolerant to water stress Sensitive to high temperatures Low initial quality	4	2	2
3	Tolerant to water stress Low initial quality	6	2	4
4	Sensitive to low or high temperatures Tolerant to water stress Low initial quality	8	5	3
5	None	17	11	6
6	Tolerant to low temperature Sensitive to water stress High initial quality	6	5	1
7	Tolerant to low temperature Sensitive to water stress Sensitive to high temperatures High initial quality	2	0	2

## Supplementary Materials

The following are available online at https://www.mdpi.com/2223-7747/9/3/386/s1, Table S1: Genotypes characteristics and constants.
